# Analytical Processing of Binary Mixture Information by Olfactory Bulb Glomeruli

**DOI:** 10.1371/journal.pone.0029360

**Published:** 2011-12-20

**Authors:** Max L. Fletcher

**Affiliations:** Department of Neurobiology and Anatomy, University of Texas Medical School, Houston, Texas, United States of America; Center for Genomic Regulation, Spain

## Abstract

Odors are rarely composed of a single compound, but rather contain a large and complex variety of chemical components. Often, these mixtures are perceived as having unique qualities that can be quite different than the combination of their components. In many cases, a majority of the components of a mixture cannot be individually identified. This synthetic processing of odor information suggests that individual component representations of the mixture must interact somewhere along the olfactory pathway. The anatomical nature of sensory neuron input into segregated glomeruli with the bulb suggests that initial input of odor information into the bulb is analytic. However, a large network of interneurons within the olfactory bulb could allow for mixture interactions via mechanisms such as lateral inhibition. Currently in mammals, it is unclear if postsynaptic mitral/tufted cell glomerular mixture responses reflect the analytical mixture input, or provide the initial basis for synthetic processing with the olfactory system. To address this, olfactory bulb glomerular binary mixture representations were compared to representations of each component using transgenic mice expressing the calcium indicator G-CaMP2 in olfactory bulb mitral/tufted cells. Overall, dorsal surface mixture representations showed little mixture interaction and often appeared as a simple combination of the component representations. Based on this, it is concluded that dorsal surface glomerular mixture representations remain largely analytical with nearly all component information preserved.

## Introduction

Olfaction is considered a synthetic sense, with odors being perceived as unique, individual perceptions. This is most apparent in the case of mixtures, where identification of individual components of a mixture is difficult, especially as the number of components is increased [1]. Thus, while naturally occurring odorants are often composed of hundreds of components, the quality of many of the individual components is often not perceived in the mixture, a phenomenon known as mixture interaction. The degree to which mixture interaction occurs presumably depends on how the neuronal representations of the components influence each other as they pass through the different stages of olfactory processing.

While evidence exists for interactions at the receptor level [2]–[7] recent imaging experiments suggest that olfactory sensory neuron input patterns onto the olfactory bulb (OB) are largely analytical with little component interaction [8]–[10]. However, more frequent and complex interactions have been observed in deeper levels, especially in olfactory output neurons [10]–[15] and olfactory cortex [12], [14], [16].

The majority of these interactions were incidences of mixture suppression, in which the response to the mixture was less than the response to the strongest component. The mechanism for this, while partially peripheral, most likely involves suppression of bulbar responses via lateral inhibition. Within the bulb are two separate populations of inhibitory interneurons that could be responsible. Granule cells located deep within the bulb form reciprocal synapses with mitral/tufted (M/T) cell dendrites and mediate lateral and feedback inhibition of mitral/tufted cell output [17]–[19]. More recent work has also identified an extensive lateral inhibitory network within the glomerular layer that serves to inhibit M/T cell responses to sensory neuron input in a center-surround fashion [20], [21]. These networks work to shape postsynaptic responses to sensory input and could serve as the initial site of mixture interactions with the central olfactory system.

To date, it is not clear if glomerular level mitral/tufted cell mixture representations of OSN input are analytic or serve as the initial site for synthetic processing with the olfactory system. To address this question, OB glomerular mixture responses were examined using a transgenic mouse line that expresses the GFP-based calcium indicator GCaMP2 in OB M/T cells [22]. Using these mice, dorsal surface glomerular representations of binary mixtures were compared to the representations of each of the mixture components. Overall, it was found that OB mixture representations appear as a simple combination of the two component maps, with very little mixture interaction observed. These results suggest that dorsal surface OB mixture representations are largely analytical in the early stages of OB processing.

## Results

**Figure 1:**
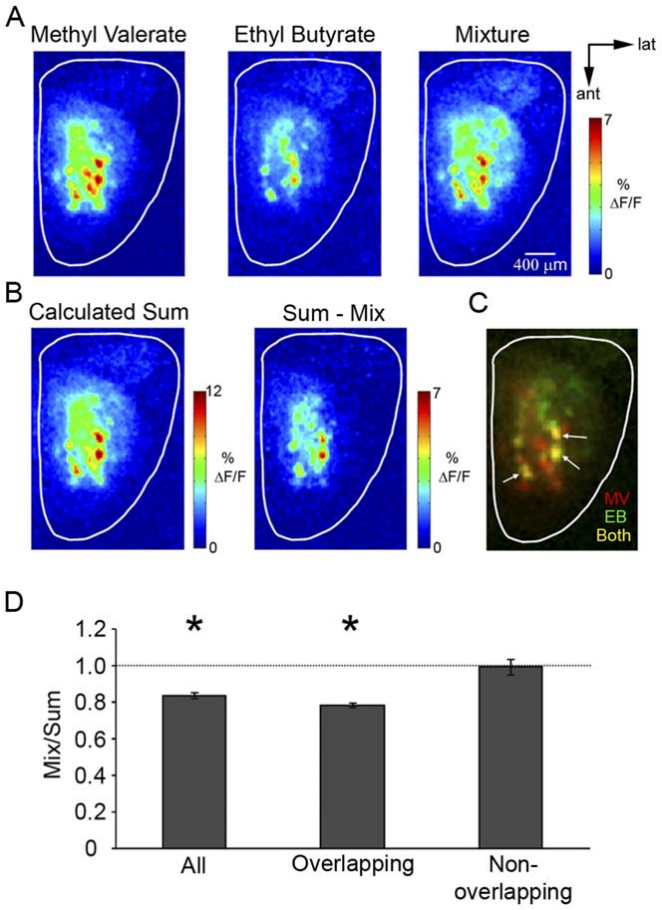
Comparison of mixture-evoked glomerular maps to the sum of the component maps. **A**, Experimentally derived responses to MV (0.25% s.v.), EB (0.25% s.v.), and their mixture displayed in pseudocolor. **B**, The calculated mixture response overestimates the mixture response as nearly all glomeruli remain. This can be seen when the mixture-evoked map is subtracted from the calculated map. **C**, Overlay of component representations expressed in different color channels to indicate glomeruli responding to one or both components (MV only: red; EB only: green; both: yellow). Glomeruli responding to both components (white arrows) are the most often overestimated by the calculated sum. **D**, Mean mixture-to-sum responses for all glomeruli, those that responded to both components (overlapping glomeruli), and those that only responded to one component of the mixture (non-overlapping glomeruli). Asterisks denote significant difference.

Odor-evoked GCaMP2 activity patterns were observed across the dorsal surface of the OB (Figure 1A). To compare mixture-evoked responses to that of each of its components, both mixture and component response maps were obtained from each animal. In all cases, mixture response maps contained all the glomeruli present in each of the component maps. In no case were new glomeruli observed in the mixture response maps that were not present in either of the component maps.

Although the number of glomeruli did not change, the intensity of individual glomeruli did. Based on a classification system described previously, glomerular responses to binary mixtures were placed into one of three groups: suppression, hypoadditivity, or synergy [4], [12], [23] (See Materials and Methods). Overall, 83.7% of 343 glomeruli displayed hypoadditive mixtures responses to the odorants used in this study. Only 5.7% of the glomeruli showed mixture suppression and 10.5% showed mixture addition.

Experimentally derived mixture response maps were then compared to the calculated sum of the activity maps for the individual components (Figure 1B). In all cases, these predicted maps contained glomeruli that were more strongly activated as compared with the experimental mixture responses. Thus, subtraction of the experimental map from the calculated map yielded a map representing glomeruli that do not display simple summation. In almost every case (92.9% of glomeruli), these glomeruli were clearly activated by both odorants in the mixture (Figure 1C; white arrows).

The mean odor-evoked relative change in fluorescence (ΔF/F) for each glomerulus was then measured in response to the binary mixture and to each component. Similar to the odor maps, the predicted sum was calculated by summing the responses to both components. The ratio of the mixture response to the summed response was then calculated for each glomerulus. In this way, a value of 1 would indicate that the measured mixture response was identical to the calculated sum. Across all glomeruli (n = 343), the mean mixture-to-calculated sum ratio was found to be significantly less than 1 (ratio: 0.82±0.01, one sample t-test, t = −12.9, p<0.001). For further analysis, all glomeruli were divided into two groups: those that only responded to one component of the mixture (non-overlapping glomeruli) and those that responded to both components (overlapping glomeruli). For overlapping glomeruli (n = 260), the mean mixture-to-sum ratio was also found to be significantly less than 1 (ratio: 0.77±0.01, one sample t-test, t = −18.1, p<0.001). For non-overlapping glomeruli n = 87), mean mixture-to-sum ratio was not significantly different than 1 (ratio: 0.96±0.04, one sample t-test, t = −1.35, p = 0.18, ns) (Figure 1D). These data suggest that, although postsynaptic mixture-evoked glomerular activity patterns appear as a combination of the individual component activity patterns, the intensity of each glomerulus does not reflect simple summation of the each component activities.

**Figure 2:**
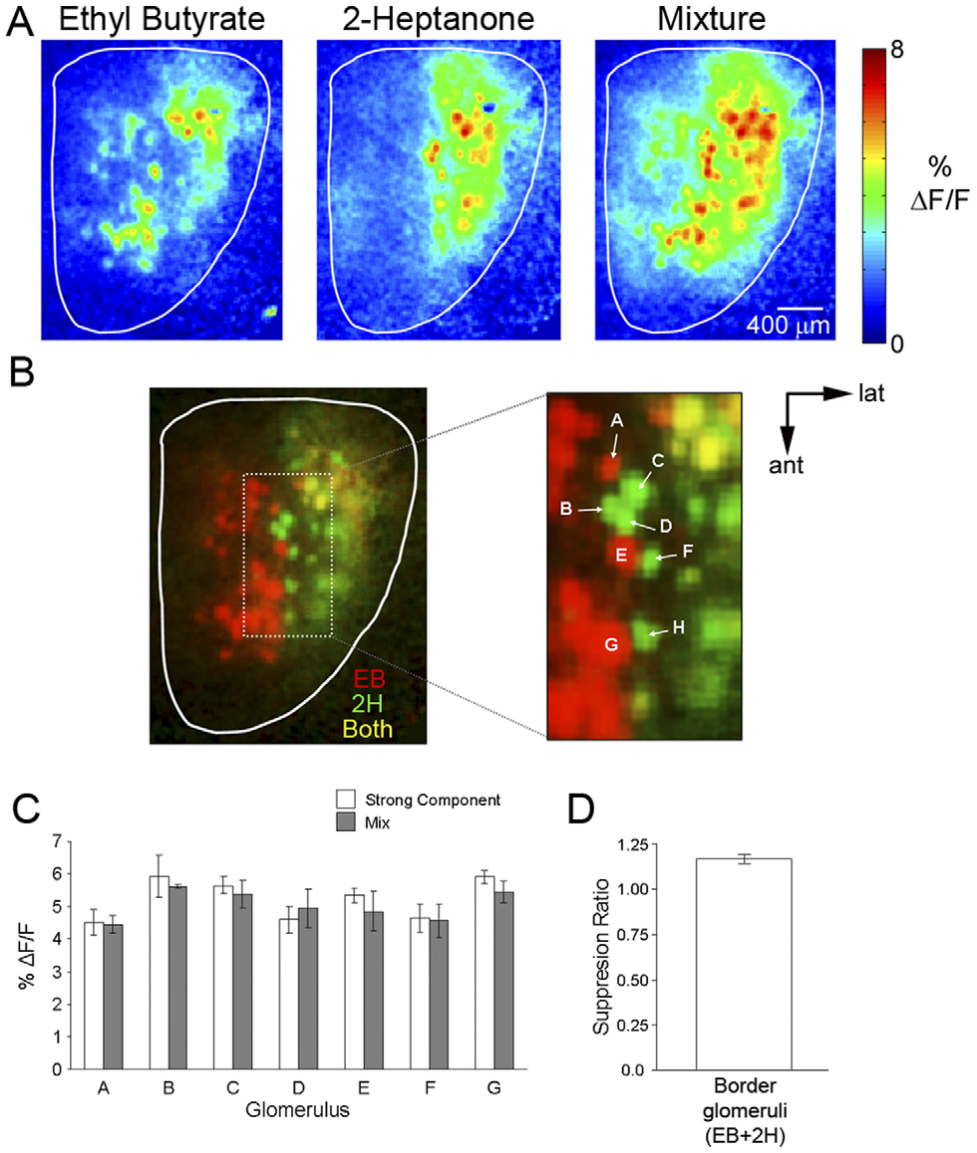
Glomerular maps of binary mixtures whose components activate neighboring glomerular areas. **A**, Glomerular maps of EB (0.5% s.v.), 2H (0.5% s.v.), and their mixture. **B**, Left: Overlay of component maps expressed in different color channels (EB only: red; 2H only: green; both: yellow). Right: Magnified image of outlined region from left showing border glomeruli. **C**, Mean strong component and mixture responses taken from glomeruli labeled in B. No significant difference was observed between the two responses for any of the labeled glomeruli. **D**, Mean suppression ratio (strong component response/mixture response) calculated from all border glomeruli for the EB+2H mixture.

According to the previous anatomical and functional studies [20], [21], [24], each glomerulus has the potential to interact and influence nearby other nearby glomeruli via lateral connections. To reveal these possible interactions, we focused on analyzing responses to odorant pairs that activated neighboring sets of different glomeruli. For example, EB activated a region of glomeruli located on the anterior-dorsal surface, while 2H activated a more lateral region of glomeruli (Figure 2A). Superimposition of the activity patterns for each odorant (EB only: red; 2H only: green; both: yellow) clearly showed a distinct zone running along the midline of the dorsal surface in which adjacent glomeruli were activated by only a single component (Figure 2B). These glomeruli, which were termed border glomeruli, could be observed not only in EB/2H combination, but also in other odorant pairs including: MV/BA; EB/BA; and MV/2H. To look for the glomerular interaction, especially suppression, we compared the intensity of the SC response to the mixture response in all of the border glomeruli (A–H glomeruli in figure 2B) (Figure 2C). For the EB/2H mixture, no significant differences between the two responses were observed in any of the border glomeruli (n = 34 glomeruli, 3 mice) with the mean suppression ratio (SR) calculated to be 1.19±0.03 (SR range: 0.92–1.65). All border glomeruli from all mixtures (n = 114) also showed no overall suppression (SR = 1.07±0.02) (Figure 2D). Finally, no overall suppression was observed in the mixture responses from all non-overlapping glomeruli for all mixtures (n = 189 glomeruli; SR = 1.09±0.02).

**Figure 3:**
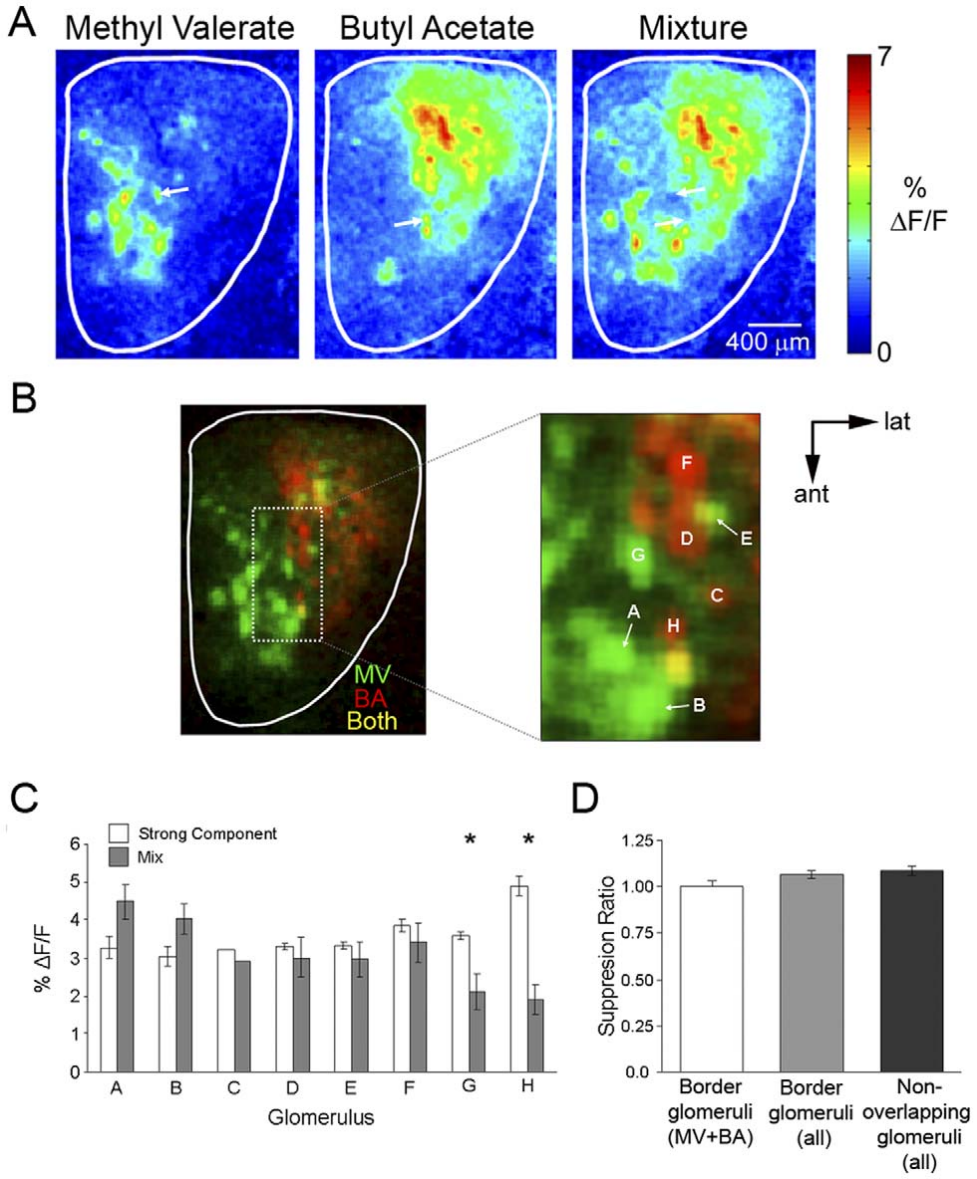
Little interaction in border glomeruli responses to binary mixtures. **A**, Glomerular response to MV (0.1% s.v.), BA (0.5% s.v.), and their mixture. Suppressed responses were observed in two border glomeruli (white arrows) when presented with the mixture. **B**, Left: Overlay of component maps expressed in different color channels (MV only: green; BA only: red; both: yellow). Right: Magnified image of outlined region from left showing border glomeruli. Glomeruli G and H are glomeruli identified in A. **C**, Mean strong component and mixture responses from glomeruli labeled in B. A seen above, a significant difference was observed between the two responses for observed for glomeruli G and H. **D**, Mean SR calculated from all border glomeruli for the MV+BA mixture only, all border glomeruli, and all non-overlapping glomeruli. Asterisks denote significant difference.

Similar results were obtained in MV/BA mixtures (Figure 3). As a group, border glomeruli showed no significant decrease in response to the mixture with an overall mean SR of 1.01±0.03 (SR range: 0.3–1.4) (Figure 3D). However, unlike the EB/2H mixtures, some individual glomeruli appeared to be suppressed by the MV/BA mixture. For example, in the case shown, both glomerulus G and H (Figure 3A, white arrows) displayed significantly reduced responses to the mixture compared to the SC (Figure 3C). Overall, this mixture suppression was observed in 16.3% of border glomeruli (n = 80) and was evident in 6 out of 9 animals presented with the MV/BA mixture. As a group, the glomeruli displaying significant mixture suppression had a mean SR of 0.65±0.04 (SR range: 0.33–0.80). No overall component preference was seen in this subset, with approximately half of the suppressed glomeruli responding to MV as the strong component. The location of these suppressed glomeruli also differed from animal to animal and were scattered across the border region. As individual glomeruli cannot be readily identified in different animals, attempts to correlate their location within the mixture maps across animals with the limited odor set used here were unsuccessful.

## Discussion

As the glomerular layer is the site of information transfer from receptor neuron to output neuron within the bulb, it provides an ideal place to investigate if the complex mixture interactions observed in mitral cells can arise from lateral processing in the early stages of the OB. With the odorants and concentrations used here, postsynaptic mixture-evoked maps appeared to reflect a simple combination of the maps evoked by each individual component with very little mixture interaction observed.

Recent studies using intrinsic optical imaging in the rodent to investigate mixture interaction at the level of OSN input into the bulb have reported that glomerular responses to binary mixtures can be predicted by the linear sum of the individual component responses [8], [25]. Similar to these studies, this study found that the mixture activated the same spatial pattern of glomeruli as the spatial combination of the component patterns. But, unlike the intrinsic imaging studies, purely postsynaptic mixture representations showed a nonlinear interaction of the components, with most glomerular mixture responses being less than their predicted response. In most cases, individual glomeruli responded to the mixture as if the strong component was presented alone. Similar nonlinear interactions have also been reported in calcium imaging studies of mixture representations at the level of OSN input into the zebra fish OB [10] and insect antenna lobe [3], [26] as well as OB glomerular odor representation modeling studies [27]. As GCaMP2-reported glomerular odor responses reflect M/T cell dendritic activity, these results suggest that the initial postsynaptic coding of odor information within the glomerular layer primarily reflects responses to OSN input.

The high incidence of hypoadditivity observed in this study also suggests that there is very little mixture interaction in the early postsynaptic stages of OB processing. These results are similar to reports of mixture responses in vertebrate olfactory sensory neurons *in vivo*, in which a majority of neuron activity displayed little interaction to binary mixtures [4], [7]. While mixture responses obtained from M/T cell single unit responses are often most similar to the responses of the strongest component [15], there have been much higher incidences of suppression reported [10], [12]–[15], [26], [28], [29]. This, together with the lack of glomerular level mixture suppression observed here suggests that mixture suppression observed at the level of M/T cell output in previous studies most likely arises from granule cell mediated lateral inhibitory activity within the deeper layers of the bulb.

Overall, the rarity of mixture suppression observed is surprising given the extensive presynaptic and postsynaptic inhibitory networks that exist in the glomerular layer [19], [20], [23], [30]. The lack of large-scale suppression, especially between border glomeruli, suggests that lateral inhibition of glomerular M/T cell dendritic tuft odor responses may not be highly prevalent, at least in the case of binary mixtures. These results fit well with studies in both fish and insects [25], [29] that suggest a two-tiered model of glomerular inhibition, in which the majority of lateral inhibition, and thus mixture component interactions, arise at the level of M/T-granule cell interactions. This brings into question the function of glomerular layer lateral inhibitory networks and the role they play in shaping M/T cell odor responses. To fully address this, future studies should be aimed at investigating the transformation of odor responses in M/T cells from the dendritic tuft to the somatic output level.

However, some evidence of mixture interaction was observed, as a small number of incidences of mixture suppression did occur. These were most frequently seen in the border glomeruli of MV/BA mixtures. It is not clear if these instances of suppression reflect postsynaptic processing by glomerular inhibitory circuits or interactions at the peripheral OSN level. A recent imaging study using the OMP-synapto-pHluorine mouse, that expresses an indicator of presynaptic OSN transmitter release, also found little mixture suppression using similar odor combinations [30]. Thus, the instances of suppression that were observed here may simply be due to interactions of the component molecules at the receptor level [4], [6], [7]. Alternatively, the lack of a large-scale mixture suppression observed here could possibly be due to the limited odor set used, as Johnson et al. [31] reported instances of glomerular level mixture suppression as measured by 2-deoxyglucose uptake in some natural odor mixtures but not in others. Finally, it is worth noting that the experiments of this study were carried out in anesthetized animals. Given that recent electrophysiological studies have demonstrated that mitral cell odor responses are often stronger in anesthetized mice compared to awake behaving mice [32], it is possible that amount of mixture suppression observed in the glomerular layer could be quite different in awake animals.

Overall, little mixture interaction was observed in olfactory postsynaptic glomerular responses to the odorants used in this study. In most cases, postsynaptic glomerular binary mixture representations appeared to be a combination of the component representations, regardless of the degree of overlap between the components. This suggests that at the glomerular level, M/T cell mixture representations are largely analytical and retain nearly all of the component information. These findings support recent behavioral studies showing that individual component odorant input patterns cannot be used to predict mixture quality [8], [33]–[35]. In conclusion, the results presented here also suggest that the majority of binary mixture interactions and subsequent synthetic perception of mixtures may not be a consequence of mixture representations interacting at the glomerular level.

## Materials and Methods

### Animals and surgery

Experiments were performed on adult transgenic mice expressing the fluorescent calcium indicator GCaMP2 under the Kv3.1 voltage-gated potassium channel promoter [36]. Mice were anesthetized with pentobarbital (50 mg/kg, i.p.) and placed in a stereotaxic apparatus situated above a heating pad to maintain body temperature. After local application of 2% lidocaine, an incision was made into the skin above the dorsal surface of the skull and the bone overlying the OB was thinned. Anesthesia was maintained throughout imaging experiments with subsequent injections of pentobarbital. Animals were allowed to breathe freely. Animal protocols (HSC-AWC-08-143) were approved by the University of Texas Institutional Animal Care and Use Committee in accordance with the NIH guidelines.

### Optical imaging

Imaging was performed using an Olympus BX50WI microscope equipped with 4x (0.28 NA) and 10x (0.3 NA) objectives. The OB was illuminated with a Polychrome II monochromator with an excitation wavelength centered at 480 nm. The G-CaMP2 fluorescence signal was band-passed filtered with a Chroma emission filter (HQ535/50) and recorded with a cooled, back-illuminated CCD camera (Redshirt Imaging) at 25 Hz with a 256×256 resolution. Image acquisition and analysis was performed using NeuroPlex software (Redshirt Imaging) and Matlab.

### Odorant presentation

Odorants were delivered using a flow-dilution olfactometer as described previously (Fletcher *et al.* 2009). Odorant were delivered for 2 seconds with an inter-stimulus interval of at least 60 seconds. Each odorant was presented separately and then together as a mixture. The final concentration of the mixture was the sum of the concentrations of its components. Odorant concentrations used were between 0.06%–1% of saturated vapor concentration. Odorants used were: propanal, butanal, pentanal, amyl acetate, butyl acetate (BA), ethyl butyrate (EB), 2-heptanone (2H), and methyl valerate (MV). Mixtures consisted of 15 different combinations of odorants and concentrations.

### Response analysis

Odor-evoked spatial activity maps were first corrected for photo-bleaching by subtracting a no-odor trial and applying a low pass spatial filter (3×3 median). The odor-evoked change in fluorescence was calculated by subtracting the average of 5 frames preceding stimulus onset from the average of 5 frames centered on the peak of the response generated by the first full respiration following odor onset. The relative change in fluorescence (ΔF/F) was calculated by dividing the odor-evoked change in fluorescence by the resting fluorescence and expressed in pseudocolor. All maps are the average of several presentations of each odorant. The predicted mixture map was created by summing the corresponding pixel values from the experimentally derived component maps. The predicted map was then subtracted from the experimentally measured response to produce a difference map.

For quantitative analysis of individual glomeruli, the odor-evoked response was calculated from the spatial average of 4 to 9 pixels located at the center of each glomerulus. Responses were averaged over several trials (2–8) to obtain a final mean ΔF/F value for each glomerulus for each odorant. Only glomeruli that displayed clear responses to least one component or the mixture were used for analysis. A glomerulus was considered to respond to an individual odorant if its mean ΔF/F response was greater than the background ΔF/F signal. Background signals were defined as the mean±2SD ΔF/F value obtained from regions containing no odor-evoked activity.

For each glomerulus, the component that elicited the smaller response was designated as the weak component (WC) and the component the elicited the larger response was designated as the strong component (SC). The predicted mixture response was calculated by summing the responses of the two components. For each glomerulus, the experimental mixture response was then divided by the predicted response. It is possible that in some cases the predicted mixture response for a given glomerulus could be beyond the maximum response amplitude for that glomerulus. To avoid any potential confounds due to saturation, all odorants were given at low concentrations (0.06–1% s.v). In anesthetized GCaMP2 mice, these concentrations elicit ΔF/F responses that are well below the saturation level of the GCaMP2 indicator. With the concentrations used in this study, the average sum of the component responses for all glomeruli was 4.6±0.1% ΔF/F. When the same odorants are delivered at high concentrations (>10%), individual glomerular responses can often be as high as 20% ΔF/F. Based on this, it is unlikely that the sum of component responses for a given glomerulus in this study would be greater than its saturation point.

To confirm this, in a subset of glomeruli in which the predicted response was larger than the mixture response, we compared the predicted (summed) mixture response to the response elicited by the strong component presented at higher concentrations than in the mixture. In 90% of the cases, higher concentrations of the strong component resulted in glomerular responses that were larger in amplitude than that of the summed responses. This suggests that at the low concentrations used here, the lack of additivity is not due to saturation of the glomerular response. However, in some cases, it is possible that saturation of the glomerular signal at lower concentrations would to an overestimation of number of glomeruli displaying mixture responses that were less than the calculated sum.

To identify mixture suppression, a suppression ratio (SR) was calculated for each glomerulus by dividing the mixture response by the strong component response. The response to a mixture was then compared to the response elicited by the strongest component. Glomeruli were then placed into one of three separate categories: suppression (mixture response<SC response), hypoadditivity (mixture response = SC response), or synergy (mixture response >SC response) [4], [12], [22]. Statistical significance was determined by t-test. Error values are reported as SE.
